# Global benchmarking of children's exposure to television advertising of unhealthy foods and beverages across 22 countries

**DOI:** 10.1111/obr.12840

**Published:** 2019-04-11

**Authors:** Bridget Kelly, Stefanie Vandevijvere, SeeHoe Ng, Jean Adams, Lorena Allemandi, Liliana Bahena‐Espina, Simon Barquera, Emma Boyland, Paul Calleja, Isabel Cristina Carmona‐Garcés, Luciana Castronuovo, Daniel Cauchi, Teresa Correa, Camila Corvalán, Emma Lucia Cosenza‐Quintana, Carlos Fernández‐Escobar, Laura I. González‐Zapata, Jason Halford, Nongnuch Jaichuen, Melissa L. Jensen, Tilakavati Karupaiah, Asha Kaur, María F. Kroker‐Lobos, Zandile Mchiza, Krista Miklavec, Whadi‐ah Parker, Monique Potvin Kent, Igor Pravst, Manuel Ramírez‐Zea, Sascha Reiff, Marcela Reyes, Miguel Ángel Royo‐Bordonada, Putthipanya Rueangsom, Peter Scarborough, Maria Victoria Tiscornia, Lizbeth Tolentino‐Mayo, Jillian Wate, Martin White, Irina Zamora‐Corrales, Lingxia Zeng, Boyd Swinburn

**Affiliations:** ^1^ Early Start, School of Health and Society University of Wollongong Wollongong New South Wales Australia; ^2^ School of Population Health University of Auckland Auckland New Zealand; ^3^ Faculty of Health Sciences National University of Malaysia Bangi Malaysia; ^4^ Centre for Diet & Activity Research, MRC Epidemiology Unit University of Cambridge Cambridge UK; ^5^ Fundación InterAmericana del Corazón–Argentina (FIC Argentina) Buenos Aires Argentina; ^6^ Nutrition and Health Research Center (CINyS) Instituto Nacional de Salud Pública (INSP) Cuernavaca Mexico; ^7^ Department of Psychological Sciences University of Liverpool Liverpool UK; ^8^ Malta College of Arts, Science and Technology (MCAST) Paola Malta; ^9^ School of Nutrition and Dietetics, University of Antioquia, Health Education and Nutrition Education Interdisciplinary Research Group (GIIESEN) Medellín Colombia; ^10^ Department of Public Health University of Malta Msida Malta; ^11^ School of Communication Diego Portales University Santiago Chile; ^12^ Institute of Nutrition and Food Technology (INTA) University of Chile Santiago Chile; ^13^ INCAP Research Center for Prevention of Chronic Diseases Institute of Nutrition of Central America and Panama Guatemala City Guatemala; ^14^ Spanish National School of Public Health Institute of Health Carlos III Madrid Spain; ^15^ School of Nutrition and Dietetics, University of Antioquia, Social and Economic Determinants of Health and Nutrition Research Group Medellín Colombia; ^16^ International Health Policy Program Ministry of Public Health Nonthaburi Thailand; ^17^ School of Nutrition University of Costa Rica San Pedro Costa Rica; ^18^ School of Biosciences, Faculty of Health and Medical Sciences Taylor's University Subang Jaya Malaysia; ^19^ Centre on Population Approaches for Non‐communicable Disease Prevention, Nuffield Department of Population Health University of Oxford Oxford UK; ^20^ School of Public Health, Faculty of Community and Health Sciences University of the Western Cape Bellville South Africa; ^21^ Nutrition Institute Ljubljana Slovenia; ^22^ Population Health, Health Systems and Innovations Human Science Research Council Cape Town South Africa; ^23^ School of Epidemiology and Public Health, Faculty of Medicine University of Ottawa Ottawa Ontario Canada; ^24^ Department for Policy in Health Ministry for Health Valletta Malta; ^25^ NIHR Oxford Biomedical Research Centre, Oxford University Hospitals NHS Foundation Trust and Centre on Population Approaches for Non‐communicable Disease Prevention, Nuffield Department of Population Health University of Oxford Oxford UK; ^26^ Pacific Research Centre for the Prevention of Obesity and Non‐communicable Diseases Fiji National University Suva Fiji; ^27^ School of Public Health University of Costa Rica San Pedro Costa Rica; ^28^ School of Public Health Xi'an Jiaotong University Health Science Center Xi'an PR China

**Keywords:** advertising, food, INFORMAS, television

## Abstract

Restricting children's exposures to marketing of unhealthy foods and beverages is a global obesity prevention priority. Monitoring marketing exposures supports informed policymaking. This study presents a global overview of children's television advertising exposure to healthy and unhealthy products. Twenty‐two countries contributed data, captured between 2008 and 2017. Advertisements were coded for the nature of foods and beverages, using the 2015 World Health Organization (WHO) Europe Nutrient Profile Model (should be permitted/not‐permitted to be advertised). Peak viewing times were defined as the top five hour timeslots for children. On average, there were four times more advertisements for foods/beverages that should not be permitted than for permitted foods/beverages. The frequency of food/beverages advertisements that should not be permitted per hour was higher during peak viewing times compared with other times (*P* < 0.001). During peak viewing times, food and beverage advertisements that should not be permitted were higher in countries with industry self‐regulatory programmes for responsible advertising compared with countries with no policies. Globally, children are exposed to a large volume of television advertisements for unhealthy foods and beverages, despite the implementation of food industry programmes. Governments should enact regulation to protect children from television advertising of unhealthy products that undermine their health.

## 1 INTRODUCTION

The marketing of unhealthy foods and beverages to children has been repeatedly identified in comprehensive evidence reviews as a negative influence on children's food knowledge, preferences, consumption, diet quality, and health.^1^, ^2^, ^3^, ^4^, ^5^, ^6^, ^7^ Therefore, restricting this marketing has been a global priority for obesity and diet‐related noncommunicable disease (NCD) prevention.^8^, ^9^, ^10^ The current World Health Organization (WHO) Global Action Plan for the prevention and control of NCDs 2013‐2020 identifies policies to reduce the impact of the marketing of unhealthy foods and beverages on children as one of 25 indicators for change in order to reduce premature mortality from NCDs by 25% by 2025.^11^ In 2010, 193 Member States endorsed Resolution WHA63.14 to restrict the marketing of food and nonalcoholic beverage products high in saturated fats, trans fats, free sugars, and/or sodium to children and adolescents.^8^ Global progress has been limited on restricting the nature and extent of children's exposure to the marketing of unhealthy foods and beverages and has been addressed primarily by industry self‐regulatory programmes.^12^, ^13^, ^14^

Globally, almost one in five people aged 5 to 19 years were overweight or obese in 2016, and the prevalence of obesity in young people has increased 10‐fold in the last 40 years.^15^ While the overall rate of increase in children's body mass index seems to have plateaued (at a high level) in high‐income countries since 2000, rates continue to increase in low‐ and middle‐income countries.^15^ The globalization of food systems has been implicated in major shifts towards poor dietary patterns and increasing NCD risk in low‐ and middle‐income countries.^16^ Globalization includes international food trade and foreign direct investment by transnational food and beverage companies that predominantly manufacture and sell unhealthy foods and beverage products. This marketing increases the desirability and normalcy of consuming these products.^16^

The International Network for Food and Obesity/non‐communicable diseases Research, Monitoring and Action Support (INFORMAS) is a global network of researchers that aims to monitor, benchmark, and support public and private sector actions to create healthy food environments and reduce obesity and NCDs.^17^ This includes monitoring and benchmarking food marketing environments over time and across place,^18^ as well as government and industry policies relating to food marketing.^19^, ^20^ Monitoring data is required throughout policy development and implementation. Initially, these data can highlight the need for policy interventions by identifying the nature and extent of children's food marketing exposures. During policy development, monitoring data can inform policy specifications, including the media, broadcast times, food products, and marketing techniques that should be restricted. Once implemented, monitoring is essential for evaluating the impact of policies on reducing children's exposures to unhealthy food and beverage marketing.

The WHO has recommended that Member States (or national governments) monitor children's exposure to, and the persuasive power of, food and beverage marketing messages.^21^ Exposure refers to the reach and frequency of contact with marketing messages, while power relates to the marketing content and design. This study had three research objectives. Firstly, given the substantial number of monitoring studies that have measured exposure and power of television food advertising to children globally,^22^ we aimed to combine these data from 22 countries to form a global overview of children's estimated exposure to advertising of unhealthy foods and beverages on television. We also sought to determine the potential impact of policy interventions, by comparing rates of television advertising for unhealthy foods and beverages to children across countries with different policy arrangements (statutory, coregulatory, or industry self‐regulation). Lastly, to identify the influence of international food trade and investment on food advertising exposures, we identified parent companies of branded food and beverage products and the penetration and frequency of advertising by parent companies (ie, the owners of subbrands or individual product brands) across markets.

## 2 METHODS

### 2.1 Procedure

Several countries (including Australia, Canada, Chile, Costa Rica, Guatemala, Malta, Mexico, New Zealand, and Slovenia) contributed data on television food advertising that had been collected using the INFORMAS standardized protocol.^23^ These data were supplemented with data from other countries, identified by a literature of studies measuring the nature and extent of food advertising on television. There was no limit on year of data collection. Included studies were those that captured television broadcasting during consecutive hours across recorded days (not limited to children's programmes) and those that included information on all food and beverage advertisements (not limited to specific food categories). Data were mostly recorded by the research team, although one dataset was purchased from a market research company (2009 UK dataset) and one was freely obtained from the national television regulator (Chile dataset). Corresponding authors were contacted and invited to participate by contributing their dataset. Datasets were eligible for inclusion if they contained sufficient information on the time each food advertisement was shown to enable calculation of the frequency of advertisements per hour, as well as sufficient detail on the advertised food and beverage products to allow reclassification using a standardized food classification system. In addition, studies needed to include at least one weekday and one weekend day, preferably randomly selected. Advertisements broadcast during school holiday periods were excluded. Datasets were cleaned by the country research teams and then sent to the lead author, who processed these for compilation.

Television food advertising policy arrangements of included countries were also captured from repositories of government and industry policies on food marketing to children^24^, ^25^ and cross‐checked with corresponding authors. Extracted policy information included the type of regulatory control (self‐regulatory codes of practice from the food industry, statutory government regulations, coregulation, or no regulation) and the year of implementation.

### 2.2 Classification of food and beverage advertisements

Advertisements included paid commercial messages that were broadcast before, during, or after television programmes. Product placements embedded within programmes were excluded, as were messages about commercial sponsorship of programmes (eg, “this program was brought to you by …”). The term “food advertisement” was used to refer to advertisements for retail food and nonalcoholic drink products, as well as advertisements for retailers themselves (supermarkets) and food service outlets (restaurants). This also included advertisements for food companies, retailers, and outlets where no specific foods or beverages were visually depicted. Food advertisements were recoded, where necessary, according to the INFORMAS protocol for television food advertising monitoring.^23^ For each advertisement, coded variables included orienting information on the placement of the advertisement (country, day, date, channel, and time of broadcast); descriptive information on the nature of the food or beverage product, company, retailer, or outlet promoted (brand/company name and description); and the use of selected persuasive marketing techniques including promotional characters (company‐owned media characters or brand‐equity mascots, third‐party licensed characters, entertainment, or sports celebrities) and premium offers (eg, competitions, rebates, and games). For advertisements where multiple products were promoted, the first product listed the description in country datasets was coded.

Advertised foods and beverages were then coded according to the WHO Regional Office for Europe Nutrient Profile Model,^26^ which was designed for the purpose of restricting the marketing of foods and beverages to children (no age range given). The model differentiates products into 16 food and four beverage categories and designates these as recommended to be “not‐permitted” or “permitted” to be advertised to children. Certain categories are recommended to not be permitted to be marketed to children regardless of their nutritional composition. These include chocolate and confectionery, cakes and sweet biscuits, juices, and energy drinks. Conversely, unprocessed meat and fish and fresh/frozen fruit and vegetables are recommended to be marketed without restriction. For other categories, threshold criteria per 100 g/mL for total fat, saturated fat, trans fat, total sugar, added sugar, nonsugar sweeteners, salt, and/or energy apply. Notably, advertisements for coffee, tea, nutritional supplements, baby food, and toddler formula are not covered by the nutrient profiling model, and these were identified separately. Advertisements for food companies, retailers, and outlets that do not promote specific food or beverage products are also not covered by the model and were similarly identified separately.

Advertisements were identified as being broadcast during children's peak television viewing times or during other viewing times. For the purposes of this study, peak viewing times were defined as the top five hour timeslots based on the maximum child audience of the day for each country, separately for weekdays and weekend days. The age of “children” varied between countries, depending on the definitions used in available audience data (see Table 1). Contributing authors from each country identified children's television audience data from available sources, reflecting average daily viewing patterns as close to the year of data collection as possible. Children's peak viewing times, children's age groups, and the source of audience data for each country are provided in Table S1.

**Table 1:** Recorded television sample description, by country and region

Country	Year of Data Collection	Age Definition for “Children,” y^a^	Channels Sampled	Transmissions on Weekdays (WD) and Weekend Days (WE)^b^	Hours per Day	Total Hours Recorded
Asia Pacific						
American Samoa	2012		3	WD: 5; WE: 1	15	90
Australia	2011	5‐12	3	WD: 6; WE: 6	17	204
China	2012	5‐12	5	WD: 6; WE: 6	16	192
Fiji	2010		1	WD: 2; WE: 2	15	60
Malaysia	2013	4‐14	3	WD: 6; WE: 6	16	192
New Caledonia	2012		1	WD: 2; WE: 2	15	60
New Zealand	2015	5‐13	3	WD: 12; WE: 12	17	408
Samoa	2012		2	WD: 4; WE: 0	15	60
Thailand	2014	<15	4	WD: 10; WE: 4	5 WD, 9 WE	344
Tonga	2012		1	WD: 2; WE: 2	5	20
Africa						
South Africa	2017	<18	4	WD: 20; WE: 8	14	392
Central and South America						
Argentina	2013/14	<18	8	WD: 47; WE: 45	16	1472
Chile	2016	<18	8	WD: 79; WE: 33	18	2016
Colombia	2012	<18	2	WD: 3; WE: 1	16	128
Costa Rica	2016	4‐11	4	WD: 16; WE: 16	18	576
Guatemala	2016	3‐11	6	WD: 24; WE: 24	18	864
Mexico	2015	<18	4	WD: 15; WE: 9	17	408
Europe						
Malta	2013	12‐18	7	WD: 35; WE: 14	15	735
Slovenia	2016	4‐9	5	WD: 25; WE: 20	18	810
Spain	2012	4‐16	5	WD: 25; WE: 10	16	552
UK (#1)	2008	4‐15	12	WD: 10; WE: 7	16	272
UK (#2)	2009	4‐15	12	WD: 60; WE: 10	16	1120
North America						
Canada	2017	2‐11	3	WD: 6; WE: 6	18	216

a Definition used to select most popular channels for children and peak viewing times.

b Refers to the total number of WD/WE days recorded across different channels, not all channels sampled on all days.

Food and beverage advertisements were also classified according to the parent company of promoted products, supermarkets, and restaurants. Parent companies of product brands were identified through local brand webpages and verified on parent company websites. This parent company information was cross‐checked against a wider web search to identify any changes in acquisition since the time of television data capture and any company subsidiaries, where particular product brands were owned by different parent companies in different countries. This web search included http://Bloomberg.com (equity trading platform) and/or Euromonitor (market research database).

### 2.3 Analyses

Data were compiled into SPSS for Windows version 21 (IBM Corp, Armonk, New York). Given the variations in advertising between weekdays and weekend days, to derive estimates from combined weekday and weekend day data, data were weighted to take account of the unequal probabilities of selection and the number of weekdays and weekend days in the sample frame for each country. The primary indicators of interest were the frequency of total food and beverage advertisements and food and beverage advertisements that should not be permitted broadcast overall and during children's peak viewing times. Advertising frequency was calculated as the mean number of advertisements per hour, per channel for each country. Differences in the frequency of advertising for foods and beverages that should not be permitted between children's peak viewing times and other viewing times were examined using paired sample t tests. Statistical significance was accepted at the level of *α* = 0.05. The frequency of advertisements for foods and beverages that should not be permitted during children's peak viewing times was compared against the regulatory arrangements in countries (government statutory regulation and/or industry code or no regulation) using one‐way analysis of variance (ANOVA) with Scheffe post hoc tests. The overall frequency of advertising across all countries was identified by parent company and the number of markets in which the top advertising companies advertised.

## 3 RESULTS

### 3.1 Sample description

Twenty‐three research groups from 22 countries contributed data for this study. The final compiled dataset spanned countries from the Asia Pacific region, Africa, Central and South America, Europe, and North America (Table 1). The total included broadcast time was 11 191 hours (mean = 486.6, SD 499.8). Most countries captured television for the majority of the day across the recording period (14‐18 h), with the exception of Tonga where data were only captured from 4:00‐9:00 pm daily and Thailand where data were captured from 3:00‐8:00 pm on weekdays and 6:00‐10:00 am and 3:00‐8:00 pm on weekend days.

### 3.2 Overall food and beverage advertising by country and region

Across countries that also captured non–food advertisements (N = 15), 23% of all television advertisements were for food or beverage products (Table 2). Overall, there were four times more advertisements for foods and beverages that would not be permitted to be advertised to children using the WHO European Region nutrient profiling model than for foods and beverage that would be permitted to be advertised (2.4 per hour vs 0.6 per hour).

**Table 2:** Average frequency of food and beverage advertising, applying the WHO Regional Office for Europe nutrient profiling model

	% All Ads for Food^a^	Average Frequency of Food Ads Ads/h/Channel (SD)			
		All Food^b^	Permitted	Not‐permitted	Ratio Permitted:Not‐permitted
Asia Pacific					
China	24	6.5 (5.8)	1.3 (2.0)	3.3 (3.7)	1:3
Australia	19	6.0 (3.2)	0.9 (1.1)	3.8 (2.6)	1:4
New Zealand	17	4.7 (3.7)	1.0 (1.1)	2.8 (2.6)	1:3
Thailand	42	3.6 (7.4)	0.0 (0.2)	2.3 (5.0)	1:58
Malaysia	15	3.2 (3.6)	0.1 (0.3)	2.4 (2.8)	1:24
Tonga^c^	–	2.7	0.0	1.8	No permitted food ads
Fiji^c^	–	0.9	0.2	0.5	1:3
Samoa^c^	–	0.9	0.2	0.4	1:2
New Caledonia^c^	7	0.3	0.1	0.1	1:1
American Samoa^c^	–	0.4	0.0	0.3	No permitted food ads
Africa					
South Africa	30	4.6 (4.2)	0.7 (1.0)	2.7 (2.8)	1:4
Central and South America					
Chile	16	2.5 (3.0)	0.6 (1.2)	1.6 (2.1)	1:3
Mexico	–	5.1 (5.6)	0.8 (1.4)	3.9 (4.4)	1:5
Colombia	21	5.3 (4.5)	0.9 (1.3)	3.9 (3.6)	1:4
Costa Rica	21	3.4 (3.2)	0.3 (0.6)	2.2 (2.4)	1:7
Guatemala	20	3.2 (3.1)	0.4 (0.9)	1.9 (2.2)	1:5
Argentina	18	2.8 (3.4)	0.2 (0.6)	2.2 (2.6)	1:11
Europe					
Spain	23	7.3 (5.0)	1.5 (1.8)	5.2 (3.5)	1:3
Slovenia	30	5.3 (6.9)	1.0 (1.7)	2.8 (3.8)	1:3
UK (2008)	–	3.1 (2.9)	0.6 (1.0)	1.9 (2.0)	1:3
UK (2009)	–	3.0 (3.1)	0.7 (1.0)	1.6 (1.9)	1:2
Malta	27	2.3 (3.4)	0.7 (1.3)	1.5 (2.6)	1:2
North America					
Canada	25	10.9 (6.9)	0.8 (1.5)	9.7 (5.9)	1:12
Overall	23	3.7 (4.4)	0.6 (1.2)	2.4 (3.1)	1:4

a Data only provided for countries capturing non–food advertisements.

b ^“^All food” includes advertisements for coffee, tea, nutritional supplements, baby food, and toddler formula. In addition, it also covers advertisements for food companies, retailers, and outlets that do not promote specific food products.

c Standard deviation cannot be calculated as data were not available by hourly timeslot but aggregated across multiple hours.

There was an average of 0.3 ads per hour (SD = 0.9) for foods and beverage that are not considered as part of WHO nutrient profiling model (2119 ads across the sample). This included coffee, tea, supplements, and baby food and toddler or follow‐up formula. Overall, 3% of food advertisements were for toddler formulas, although this was markedly higher in some countries, including China where these products comprised 21% of food ads, Guatemala (11%), Malaysia (8%), and Costa Rica (7%).

Further, there was an average of 0.3 ads per hour (SD = 0.9) for food companies or brands (no specific foods depicted), which could not be classified by the nutrient profiling model given the range of food and beverage products that the companies manufactured or sold (2206 ads across the sample). This frequency was fairly consistent across countries, ranging from 0 food company brand ads per hour in Malta to 1.2 (SD = 2.0) in Slovenia. Almost all of these types of advertisements (80%) were for supermarkets, where only the store was advertised and not food products, and a further 14% were for fast food restaurants without products advertised.

The most frequently advertised food and beverage categories overall were the following: “other” beverages (including carbonated soft drinks, mineral water, and flavoured waters) (15%; 81% should not be permitted); chocolate and confectionery (13% of all food ads; 100% should not be permitted); ready‐made food and dishes (12%; 76% should not be permitted); breakfast cereals (9%; 87% should not be permitted); and cakes, biscuits, and pastries (7%; 100% should not be permitted) (Table 3).

**Table 3:** Top five food and beverage categories advertised by country, using WHO Regional Office for Europe nutrient profiling model^a^

Country	Most Frequently Advertised Products (% Advertisements)				
	1st	2nd	3rd	4th	5th
Asia Pacific					
American Samoa	Ready‐made food & dishes (75%)	Other beverages (25%)	–	–	–
Australia	Ready‐made food & dishes (45%)	Chocolate & confectionery (12%)	Savoury snacks (6%)	Sauces, dips, dressings (6%)	Breakfast cereals (6%)
China	Other beverages (30%)	Yoghurts, sour milk, similar foods (12%)	Milk drinks (12%)	Chocolate & confectionery (10%)	Ready‐made food & dishes (8%)
Fiji	Other beverages (31%)	Ready‐made food & dishes (25%)	Breakfast cereals (11%)	Juices (8%)	Chocolate & confectionery (6%)
Malaysia	Cakes, biscuits, pastries (20%)	Ready‐made food & dishes (16%)	Other beverages (14%)	Chocolate & confectionery (13%)	Milk drinks (8%)
New Caledonia	Ready‐made food & dishes (50%)	Pasta, rice & grains (43%)	Other beverages (7%)	–	–
New Zealand	Ready‐made food & dishes (22%)	Chocolate & confectionery (16%)	Fresh/frozen meat, poultry, fish (9%)	Sauces, dips, dressings (7%)	Breakfast cereals (6%)
Samoa	Yoghurts, sour milk, similar foods (29%)	Edible ices (23%)	Fresh/frozen fruit, veg, legumes (23%)	Savoury snacks (13%)	Pasta, rice & grains (7%)
Thailand	Other beverages (21%)	Ready‐made food & dishes (17%)	Chocolate & confectionery (14%)	Yoghurts, sour milk, similar foods (14%)	Savoury snacks (11%)
Tonga	Other beverages (65%)	Edible ices (20%)	Ready‐made food & dishes (15%)	–	–
Africa					
South Africa	Breakfast cereal (15%)	Other beverages (15%)	Ready‐made food & dishes (14%)	Chocolate & confectionery (13%)	Sauces, dips, dressings (8%)
Central and South America					
Argentina	Other beverages (36%)	Chocolate & confectionery (18%)	Savoury snacks (9%)	Yoghurts, sour milk, similar foods (8%)	Sauces, dips, dressings (6%)
Chile	Other beverages (21%)	Fresh/frozen meat, poultry, fish (11%)	Chocolate & confectionery (13%)	Processed meat, poultry, fish (9%)	Milk drinks (8%)
Colombia	Other beverages (34%)	Edible ices (9%)	Cakes, biscuits, pastries (8%)	Chocolate & confectionery (7%)	Sauces, dips, dressings (7%)
Costa Rica	Chocolate & confectionery (15%)	Breakfast cereals (12%)	Other beverages (11%)	Ready‐made food & dishes (10%)	Edible ices (9%)
Guatemala	Savoury snacks (18%)	Other beverages (16%)	Ready‐made food & dishes (10%)	Cakes, biscuits, pastries (8%)	Chocolate & confectionery (8%)
Mexico	Other beverages (17%)	Yoghurts, sour milk, similar foods (13%)	Breakfast cereals (10%)	Ready‐made food & dishes (11%)	Chocolate & confectionery (9%)
Europe					
Malta	Ready‐made food & dishes (53%)	Cakes, biscuits, pastries (6%)	Fresh/frozen meat, poultry, fish (6%)	Bread, crispbread (5%)	Chocolate & confectionery (5%)
Spain	Chocolate & confectionery (15%)	Cakes, biscuits, pastries (12%)	Yoghurts, sour milk, similar foods (13%)	Other beverages (9%)	Ready‐made food & dishes (8%)
Slovenia	Chocolate & confectionery (24%)	Fresh/frozen meat, poultry, fish (15%)	Processed meat, poultry, fish (9%)	Other beverages (8%)	Fresh/frozen fruit, veg, legumes (7%)
UK (2008)	Breakfast cereals (16%)	Yoghurts, sour milk, similar foods (16%)	Sauces, dips, dressings (13%)	Ready‐made food & dishes (12%)	Cheese (8%)
UK (2009)	Breakfast cereals (20%)	Ready‐made food & dishes (13%)	Yoghurts, sour milk, similar foods (11%)	Other beverages (10%)	Fats and oils (7%)
North America					
Canada	Breakfast cereals (31%)	Chocolate & confectionery (22%)	Cakes, biscuits, pastries (14%)	Ready‐made food & dishes (13%)	Cheese (9%)
Overall	Other beverages (15%)	Chocolate & confectionery (13%)	Ready‐made food & dishes (12%)	Breakfast cereals (9%)	Yoghurts, sour milk, similar foods (7%)

“Other” beverages include carbonated soft drinks, mineral water, and flavoured waters.

a Excludes advertisements for company brands only and non‐applicable products (coffee, tea, supplements, baby food, and toddler formula).

### 3.3 Food and beverage advertising during children's peak viewing times

Overall, for those countries where children's television audience data were available (N = 12), the mean frequency of food and beverage advertisements that should not be permitted was significantly higher during children's peak viewing times compared with other viewing times (Table 4). These timeslots when the highest number of children was watching television were typically during the late evening, up to 10:00 pm or later in most countries (Table S1). The frequency of food and beverage advertisements that should not be permitted was around 35% higher during peak viewing times overall compared with other viewing times. In countries where the frequency was significantly higher in peak viewing times, the frequency of food and beverage advertising that should not be permitted was between 48% higher (Australia) to 85% higher (Chile).

**Table 4:** Average frequency of not‐permitted food and beverage advertising by children's peak and nonpeak viewing times (top five hourly timeslots)

Country	Not‐permitted Food Ads Ads/h/Channel (SD)		
	Peak Viewing Times	Other Viewing Times	t test
Canada	13.4 (5.6)	8.2 (5.5)	−4.3***
Australia	4.9 (2.7)	3.3 (2.3)	−2.6**
Spain	4.5 (3.0)	5.2 (3.5)	ns
New Zealand	4.0 (2.9)	2.5 (2.5)	−3.4***
Colombia	4.0 (3.6)	3.9 (3.6)	ns
Slovenia	3.6 (4.1)	2.6 (3.7)	ns
Malaysia	3.5 (3.4)	2.0 (2.4)	−2.2*
South Africa	3.1 (2.4)	2.7 (3.0)	ns
Costa Rica	3.0 (2.3)	2.0 (2.0)	−3.2**
Guatemala	2.5 (2.5)	1.6 (2.0)	−3.6***
Chile	2.4 (2.4)	1.3 (1.8)	−7.6***
Malta	1.7 (2.6)	1.4 (2.1)	ns
Overall	3.1 (3.4)	2.3 (3.1)	−6.7***

* *P* < 0.05.

** *P* < 0.01.

*** *P* < 0.001.

### 3.4 Persuasive marketing techniques

Most countries, with the exception of the five Pacific Island countries, Chile and the United Kingdom (2009) had comparable information on the use of promotional characters and premium offers in food advertisements. Of these 16 countries, 30% of food and beverage advertisements contained promotional characters, and of these, three‐quarters (73%) were for products that would not be permitted to be advertised according to WHO nutrient profiling. Overall, 21% of food and beverage advertisements contained premium offers, and 67% of these were for products that should not be permitted. The rate of food and beverage advertisements containing promotional characters was significantly higher during children's peak viewing times (1.8 food ads per hour vs 1.5 during nonpeak times; *t* = −3.6, *P* = 0.0001). The rate of food and beverage advertisements containing premium offers was also significantly higher during children's peak viewing times compared with nonpeak times (1.4 vs 1.2; *t* = −3.1, *P* = 0.002).

### 3.5 Policy evaluation

Table 5 shows the television food advertising policies in each country at the time the data were collected, in descending order of the frequency of food advertisements that should not be permitted per hour during children's peak viewing times. Captured policies include statutory regulations, coregulatory approaches, and industry self‐regulation. At the time of data collection, only five countries had enforced government statutory regulations: Australia, Mexico, South Africa, Thailand, and the United Kingdom. Chile introduced government statutory regulations in June 2016, just following the data collection period. Six countries had food industry codes of practice for responsible food marketing to children in place at the time of data collection: Canada (excluding Quebec), Colombia, Malaysia, New Zealand, Slovenia, and Spain.

**Table 5:** Frequency of television food and beverage advertising by policy arrangement

Country	Type of Regulations	Year of Policy Start	Year of TV Data Capture	Frequency Not‐permitted Food Ads in Peak Viewing Times (SD)	Ratio Permitted:Not‐permitted in Peak Times
Coregulation/Govt regulation					
Australia	Industry code	2009	2011	4.9 (2.7)	1:6
	& Govt regulation	2009			
South Africa	Industry code	2009	2017	3.1 (2.4)	1:3
	& Govt regulation	2014			
Mexico	Govt regulation	2014	2015	Peak viewing times N/A	
Thailand	Industry code	2008	2014	Peak viewing times N/A	
	& Govt statutory regulation	2008			
UK	Govt regulation	2007 (full implementation 2009)	2008/09	Peak viewing times N/A	
Total				3.4 (2.4)	1:4
Industry code					
Canada (excl Quebec)	Industry code	2008	2017	13.4 (5.6)	1:8
Spain	Industry code	2005	2012	4.5 (3.0)	1:4
New Zealand	Industry code	2010	2015	4.0 (2.9)	1:3
Colombia	Industry code	2013	2012	4.0 (3.6)	1:3
Slovenia	Industry code	2009	2016	3.6 (4.1)	1:3
Malaysia	Industry code	2013	2013	3.5 (3.4)	1:36
Total				3.8 (4.4)***	1:4
No policy					
Costa Rica	No policy		2016	3.0 (2.3)	1:8
Guatemala	No policy		2016	2.5 (2.2)	1:5
Chile^a^	No policy		2016 (April‐May)	2.4 (2.4)	1:3
Malta	No policy		2013	1.7 (2.6)	1:2
Argentina	No policy		2013/14	Peak viewing times N/A	
China	No policy		2012	Peak viewing times N/A	
Pacific Island	No policy		2010	Peak viewing times N/A	
Total				2.6 (2.5)***	1:4

*** *P* < 0.001.

a Government regulations introduced in Chile in June 2016, after the period of data collection.

There was a significant difference in the frequency of food and beverage advertising that should not be permitted during children's peak viewing times across countries with different policy arrangements (*F*_2,1122_ = 13.91, *P* < 0.001). Post hoc comparisons using the Scheffe test indicated that the frequency of food and beverage advertising that should not be permitted was significantly higher during children's peak viewing times in countries with voluntary food industry self‐regulatory programmes compared with countries with no policy (3.8 advertisements per hour vs 2.6).

### 3.6 Food advertising by parent company

Table 6 presents the parent companies contributing to at least 1% of overall food and beverage advertisements across the sample. Of the 43 306 food and beverage advertisements captured across the 23 datasets from 22 countries, the top 10 advertising companies contributed to one‐third (34%) (Table 6). These companies also contributed to 40% of all food advertisements that should not be permitted. Nine of these top 10 advertising companies were present across most markets.

**Table 6:** Advertising parent companies contributing to 1% or more of overall food and beverage advertisements

Parent Company	Food Company Type (Manufacturer, Retailer, Restaurant)	Country of Headquarters	Total Food Adsn (% Contribution)	Not‐permitted Food Adsn (% Contribution)	Number of Countries with Ads from Company (N out of 22)
Coca‐Cola Company	Manufacturer	USA	2010 (4.6)	1853 (6.6)	20
Kellogg Company	Manufacturer	USA	1623 (3.7)	1599 (5.7)	13
Nestle S.A	Manufacturer	Switzerland	2342 (5.4)	1289 (4.6)	16
PepsiCo, Inc	Manufacturer	USA	1397 (3.2)	1276 (4.5)	15
Danone	Manufacturer	France	1852 (4.3)	1185 (4.2)	14
Mondelez International, Inc	Manufacturer	USA	897 (2.1)	858 (3.0)	15
Unilever Group	Manufacturer	UK	1381 (3.2)	844 (3.0)	15
McDonald's Corporation	Restaurant	USA	1518 (3.5)	826 (2.9)	17
General Mills, Inc	Manufacturer	USA	912 (2.1)	800 (2.8)	5
Mars, Inc	Manufacturer	USA	784 (1.8)	669 (2.4)	13
Grupo Arcor S.A.	Manufacturer	Argentina	601 (1.4)	601 (2.1)	2
Yum! Brand, Inc	Restaurant	USA	710 (1.6)	597 (2.1)	14
Ferrero Group	Manufacturer	Italy	529 (1.2)	529 (1.9)	8
Fonterra Cooperative Group	Manufacturer	New Zealand	629 (1.5)	519 (1.8)	6
Restaurant Brands International Inc.	Restaurant	Canada	553 (1.3)	483 (1.7)	8
Post Holdings, Inc	Manufacturer	USA	542 (1.3)	421 (1.5)	2
Agrokor d.d	Manufacturer & Retailer	Croatia	689 (1.6)	262 (0.9)	1
Wal‐Mart Stores, Inc	Retailer	USA	539 (1.2)	148 (0.5)	7
Lidl Slovenija D.O.O. K.D.	Retailer	Slovenia	462 (1.1)	145 (0.5)	1

## 4 DISCUSSION

This study provides a global representation of children's exposure to food and nonalcoholic beverage advertising on television, the nature of this advertising in terms of the promoted products, and the techniques used that have a strong persuasive appeal, the main advertising companies, and the relationship of advertising to government regulation and industry self‐regulatory programmes. We identified three major findings: (1) that unhealthy foods and beverages were promoted four times more than healthier foods and beverages and the rate of unhealthy food and beverage advertisements was even higher during peak viewing times for children; (2) that the bulk of food and beverage advertisements derive from a small number of transnational companies; and (3) that existing regulatory arrangements in countries do not appear to have created more favourable/healthy television food advertising environments compared with countries without any such policies. These key points are addressed below.

We identified that, globally, rates of food and beverage advertisements that should not be permitted were 35% higher during children's peak viewing times overall compared with nonpeak viewing times and were significantly higher during children's peak viewing times in Chile (85%), Malaysia (75% higher), Canada (77%), Guatemala (56%), New Zealand (60%), Costa Rica (50%), and Australia (48%). Of these countries, at the time of data capture, only Australia had government statutory regulations in place to restrict television advertising of unhealthy food and beverages to children. It was largely these advertisements for unhealthy foods and beverages that contained persuasive elements that are appealing to children^27^ and these persuasive techniques were also most frequently used during children's peak viewing times. These findings are aligned with previous studies measuring children's television food advertising exposures, which similarly found the highest rates of advertising for unhealthy foods and beverages during broadcast periods when the greatest number of children was watching.^28^, ^29^, ^30^

The top five most frequently advertised food and beverage categories were “other beverages” (including carbonated soft drinks, mineral water, and flavoured waters), chocolate and confectionery, ready‐made food and dishes, breakfast cereals, and cakes, biscuits, and pastries; the vast majority of which were unhealthy versions that exceeded the WHO Regional Office for Europe's nutrient profiling model's threshold criteria for fat, sugar, sodium, and/or energy. Across all viewing times, the rate of advertisements for foods and beverages that should not be permitted far exceeded that of permitted foods and beverages by a factor of four to one. This ratio varied across countries. In Thailand and Malaysia, for example, where there were almost no permitted food or beverage advertisements, there were 58 and 24 advertisements for unhealthy foods and beverages for each single advertisement for healthier food or beverage broadcast, respectively, as defined by the WHO Regional Office for Europe nutrient profiling model. This ratio of advertising for healthy to unhealthy foods and beverages clearly depicts a media environment that is saturated by unhealthy choices. In other countries, there were at least some food or beverage advertisements that should be permitted. For example, in New Zealand, around 9% of food advertisements overall were for fresh meat and 11% were for healthy oils, while in Malta, 3% of food advertisements were for fresh fruits and vegetables.

In our sample, one‐third of all food and beverage advertisements were derived from just 10 companies globally. All of these companies were transnational corporations, which had a combined market value of more than US$994 billion in 2017.^31^ This suggests the colossal economic power of these food and beverage manufacturing and retail industries and their potential to influence country‐level government policies affecting the production, distribution, and promotion of their products.^32^ Strong industry resistance to government regulations to limit the sale and promotion of unhealthy products has been documented.^13^, ^33^ Retaliatory techniques have included sponsoring and disseminating reports to deny the need for, or impact of, regulation; developing alliances with civil society organizations to promote physical activity interventions to control obesity; building consensus in agreement with industry's agenda; and focusing on the potential for regulations to have detrimental effects to economy and trade, including loss of jobs.^34^ Further, all 10 of these top advertising companies were signatories to the International Food and Beverage Alliance (IFBA) global commitment for responsible marketing, which since 2009 has pledged to only advertise “better‐for‐you” products to children younger than 12 years.^35^ Despite this, these companies disproportionately advertised unhealthy products compared with healthier products across all broadcast times.

The prominence of advertisements by transnational food companies across our sample highlights the penetration of foreign direct investment in the 22 countries examined in this study. Foreign direct investment refers to the investment by an enterprise in one country into an enterprise in another, whereby the foreign enterprise becomes a foreign affiliate of the parent company.^16^ Hawkes previously identified that an increasing proportion of foreign direct investment is now entering developing and transition markets, including in Latin America, Asia, and Central and Eastern Europe.^16^ This is commensurate with our findings on the contribution of transnational company‐owned product advertising in these markets. Food and beverage products owned by these transnational corporations are typically highly processed, energy dense, and high in sugar, salt, and saturated or trans fats.^36^ Such foods are hyper‐palatable, shelf‐stable, and value‐added, all leading to their high profitability for food companies. The heavy marketing of these products serves to increase their desirability and normality and, by targeting children, builds brand loyalty that can ensure lifelong product purchases.^37^

This study also indicates the ineffectiveness of existing voluntary food industry codes of practice for responsible food marketing to children, including the IFBA commitment. Data were only sourced for a single time point for each country, with the exception of the United Kingdom; hence, it is not possible to determine if industry food marketing policies led to any changes in children's exposure to marketing of unhealthy foods and beverages since their introduction. However, countries that had industry self‐regulatory codes on food marketing to children in place at the time of data collection had significantly higher rates of advertising for unhealthy foods and beverages during children's peak viewing times compared with those countries without any industry or government policies at all. This is aligned with other systematic review evidence showing that industry self‐regulation has not been effective in reducing children's exposures to unhealthy food marketing.^38^ Limitations of industry codes of practice for responsible marketing have been well documented.^13^, ^14^, ^39^ Such codes have limited impact because of their voluntary adoption, variation in applications across countries, inadequate or vague definitions for when and where food marketing to children can occur, and permissive nutrient criteria on which to base foods deemed acceptable to be promoted.^40^ Industry self‐regulatory codes do not apply any universal or independently developed nutrient profiling model for identifying foods that may be advertised to children, such as the WHO Regional Office for Europe model used in this study.

Related to television, most industry codes of practice only apply to programmes where the audience comprises at least 35% children, which occurs infrequently as identified from television audience measurement data.^40^ Alternatively, the definition of children's peak viewing times adopted in the current study was based on the absolute number of children watching. At the time of data capture, five countries—Australia, Mexico, South Africa, Thailand, and the United Kingdom—also had government regulations related to food marketing to children. Despite this, the frequency of advertisements for unhealthy foods and beverages during children's peak viewing times was not significantly different to countries with no policy or with an industry self‐regulatory code. Australia had one of the highest frequencies of advertising for unhealthy foods and beverages during children's peak viewing times. This is unsurprising given that these Australian regulations do not limit the types of foods that can be advertised to children but only make provisions on the use of some persuasive marketing techniques during a limited broadcast period (designated children's and pre‐school children's programmes) that does not correspond with peak viewing times. While South African regulations restrict advertising of unhealthy foods and beverages between 6:00 am and 9:00 pm daily, the rate of food advertising that should not be permitted during children's peak viewing times was comparable with the sample mean. Audience data to derive peak viewing times for children were not available for Mexico, Thailand, or the United Kingdom. More recently, governments from some of the other sample countries have introduced or are in the process of introducing statutory regulations on food marketing to children, including Chile, Canada, and Slovenia.^25^ The United States (USA) is one country for which there exists a relatively large amount of television food advertising data but which was not included in the current comparative study. Analyses of American children's exposure to television advertising for food and beverage products in the decade to 2016 have shown a 45% decrease in food‐related advertisements during children's programmes.^41^ This coincides with the introduction of the American Children's Food and Beverage Advertising Initiative in 2007. However, in 2016, children viewed 26% more food‐related advertisements than in 2007, given that the rate of food‐related advertisements in non‐children's programming had increased. The findings from the current study and studies from the USA suggest that restricting television advertising for unhealthy food and beverage products only during designated children's programmes will have minimal impact on children's exposure given children's peak viewing times reside outside of these programmes.

In our application of the WHO Regional Office for Europe Nutrient Profile Model to the television advertising datasets, we identified a number of considerations for applying such a model in policy. This model was specifically developed for use by European countries for classifying foods and beverages that should and should not be permitted to be advertised to children.^26^ Other WHO regional offices have since produced variations of this model, such that the models reflect cultural eating patterns within global regions. Firstly, toddler or follow‐up formulas are not covered by the WHO nutrient profiling models. The models' guidelines recognize the World Health Assembly Resolution WHA39.28, which states that the practice of providing infants with specially formulated milks is not necessary. Given the large number of advertisements for toddler or follow‐up formula in some countries, the models would benefit from explicitly stating if these advertisements should or should not be permitted. Secondly, there were a small number of advertisements for fast food company brands, where these were promoted in the absence of food or beverage products. It is foreseeable that these advertisements could increase in markets that applied the nutrient profiling model, as these products are not covered by the criteria.

This study has some limitations. As noted above, the data captured were for a single point in time for each country, with the exception of the United Kingdom, and so changes over time as a result of marketing policies in most countries could not be ascertained. Further, the study analyses drew on existing data sources and as such, the time period and specifications for data collection varied across countries. Only potential exposures could be assessed, without accounting for the number of child viewers of these advertisements (ie, the advertisement reach). The age definition of children also varied across countries, which limits the comparability of findings across countries in relation to advertising during children's peak viewing times. It should be noted that the WHO Commission on Ending Childhood Obesity^9^ and the UN Convention on the Rights of the Child^42^ both specify that governments should define children as up to the age of 18 years. Lastly, contemporary marketing is characterized by integrated communications, spanning multiple channels that reinforce promotional messages. This study was limited to measuring children's potential exposure to food and beverage advertising on television only, given comparative data for other media are not available. Projections of total marketing expenditures indicate that expenditure on online media, and particularly social media advertising, will exceed that for television advertising in 2018.^43^ As such, comparative studies on children's potential exposures to online media food marketing are needed. Strengths of this study included the recoding of country datasets according to a standardized coding frame that has been adopted by INFORMAS^23^ to allow cross‐country comparisons; the application of the WHO nutrient profiling model across countries to explore the extent that this model is appropriate for classifying products as part of food marketing regulations broadly; the use of data on child television viewing patterns to discern broadcast times when the greatest number of children is exposed; and the identification of parent companies of food and beverage advertisements, an analysis that has not been undertaken previously.

## 5 CONCLUSION

Globally, children are potentially exposed to large volumes of television advertising for unhealthy foods and beverages, despite the implementation of food industry codes of practice for responsible marketing in many countries. Such policies have been ineffective in reducing exposures to this form of marketing. Across all countries, television food and beverage advertisements are predominantly for products that exceed WHO maximum thresholds for saturated fat, sodium, and/or sugar for foods and beverages that are considered appropriate to be marketed to children. Most of these advertisements derive from multinational corporations, which reach children by buying advertising slots at times when the greatest number of children is watching and by using techniques with strong persuasive appeal that are engaging to young people. Monitoring data such as those presented in this study can be used as part of evidence‐informed policymaking by identifying the broadcast periods during which the highest frequencies of advertisements occur, the persuasive techniques that are used and identifying considerations for applying nutrient profiling models.

## Supporting information

Table S1:
Children's peak viewing times defined as the top 5 hour timeslots for child audience
