# Supplementary material for: Global benchmarking of children's exposure to television advertising of unhealthy foods and beverages across 22 countries
Source: Obes Rev. 2019 Apr 11;20(Suppl 2):116–28. doi: 10.1111/obr.12840 (PMC6988129; doi:10.1111/obr.12840)
Supplement: Supplementary file 1 — Table S1: Children's peak viewing times defined as the top 5 hour timeslots for child audience [file OBR-20-116-s001.docx]

**Supplementary table 1:** Children’s peak viewing times defined as the top 5 hour timeslots for child audience

| **Country** | **Weekday peak viewing times (hours/day)** | **Weekend peak viewing times (hours/day)** | **Source of child audience data** |  |
| --- | --- | --- | --- | --- |
| Argentina | *Peak viewing times* *not available* | | |  |
| Australia | 17:00-22:00 | 17:00-22:00 | Australian Television Audience Measurement |  |
| Canada | 18:00-23:00 | 18:00-23:00 | Nielsen Media Research |  |
| Chile | 15:00-20:00 | 15:00-20:00 | Kantar Ibope Media in Chile |  |
| China | *Peak viewing times* *not available* | | |  |
| Costa Rica | 17:00 to 22:00 | 17:00 to 22:00 | IBOPE audience data in Costa Rica |  |
| Colombia | 9:00-11:00,  19:00-22:00 | 14:00-19:00 | IBOPE audience data in Colombia |  |
| Guatemala | 13:00-16:00,  18:00-20:00 | 13:00-16:00,  18:00-20:00 | Kantar IBOPE Media |  |
| Malaysia | 14:00-15:00,  18:00-22:00 | 14:00-16:00,  19:00-22:00 | Nielsen Audience Measurement |  |
| Malta | 16:00-19:00,  20:00-22:00 | 12:00-14:00, 16:00-17:00, 19:00-21:00 | Broadcasting Authority, Malta |  |
| Mexico | *Peak viewing times* *not available* | | |  |
| New Zealand | 16:30-22:00 | 9:00-10:00,  17:30-21:30 | Nielsen Audience Measurement |  |
| Slovenia | 7:00-8:00 and  18:00-22:00 | 7:00-10:00 and  19:00-21:00 | Nielsen Audience Measurement |  |
| South Africa | 14:00-19:00 | Saturdays 9:00-14:00  Sundays 15:00-20:00 | SAARF South African Audience Research |  |
| Spain | 20:00-24:00* | 20:00-24:00* | Kantar Media |  |
| Thailand | *Peak viewing times* *not available* | | |  |
| United Kingdom | *Peak viewing times* *not available* | |  |  |

* Only 4 hours (audience data does not allow further identification of 1 hour peak time slot)
